# Juvenile Nasopharyngeal Angiofibroma: A Series of 96 Surgical Cases

**DOI:** 10.1055/s-0043-1777293

**Published:** 2024-06-04

**Authors:** Alexandre Wady Debes Felippu, Erica Batista Fontes, André Wady Debes Felippu, Bruna Coelho Ellery, Ana Carolina Silveira de Oliveira, André Vicente Guimarães, Filippo Cascio, Alexandre Felippu

**Affiliations:** 1 Instituto Felippu de Otorrinolaringologia, São Paulo, SP, Brazil; 2 Hospital das Clínicas, Faculdade de Medicina, Universidade de São Paulo, Brazil; 3 Department of Otorhinolaryngology, Papardo Hospital, Messina, Italy

**Keywords:** nasopharyngeal neoplasms, angiofibroma, surgery, intranasal surgery

## Abstract

**Introduction** Juvenile nasopharyngeal angiofibroma (JNA) is a benign vascularized tumor that affects almost exclusively male adolescents. Surgery is the treatment of choice for JNA.

**Objectives** The present study is a 42-year retrospective review of a series of JNA cases treated surgically without previous embolization.

**Methods** The present is a retrospective, descriptive study based on medical records of 96 patients with JNA who underwent microscopic or endoscopic excision without previous embolization from 1978 to 2020 in a single institution. The patients were categorized according to the Andrews et al. stage, and data were collected on age, gender, tumor staging, surgical approach, affected side, and outcome.

**Results** All patients were male, with an average age of 17 years. The predominant tumor stage consisted of type II, with 52.1%. A total of 33.3% of the patients were submitted to the microscopic technique and 66.7%, to the endonasal technique. The rate of intraoperative blood transfusion was of 17.7%.

**Conclusion** The present study reinforces that resection of JNA in various stages is viable without previous artery embolization.

## Introduction

Juvenile nasopharyngeal angiofibroma (JNA) is a benign, slow-growing, highly-vascularized, uncommon tumor. 1 2 3 4 5 6 7 8 9 10 Its rate of incidence is of 1:150 thousand, which accounts for 0.05 to 0.5% of all head and neck tumors, 6 8 10 11 and can be life-threatening due to the potential risk of bleeding and intracranial invasion. 11 It affects almost exclusively male adolescents. 1 2 3 4 5 6 7 8 9 10 Although its histological features are benign, it can be locally aggressive and is associated with high persistence and recurrence rates. 11 12

The diagnosis is based on clinical and image evaluation with contrast-enhanced sequences, such as computed tomography (CT) and magnetic resonance imaging (MRI). Additional digital subtraction angiography can also be performed to assess the vascular supply of the angiofibroma; however, it is not necessary to confirm diagnosis. 12

Surgery is currently considered the treatment of choice for JNA. 2 3 4 7 8 11 12 13 14 15 16 17 Although some studies 8 18 mention the use of adjuvant radiotherapy for unresectable tumors, failure to remove the tumor completely, or extensive intracranial extension, its use is controversial. Chemotherapy, hormonal therapy, and, more recently, gamma knife radiosurgery in conjunction with surgical resection have been occasionally proposed, although with limited experience 18 19 and controversial efficacy.

Surgical resection is considered challenging due to the high vascularity. Bleeding during the operation is associated with poor visualization and severe blood loss. 7 9 Preoperative embolization is used in most surgical procedures due to the vascular nature of the tumor, and it is considered an acceptable means of decreasing intraoperative blood loss. 1 6 12 13 15 16 20 21

The present study intends to describe the experience with a series of 96 JNA cases treated in one institution: Instituto Felippu de Otorrinolaringologia (IFO), in São Paulo, Brazil.

## Methods

The present paper consists of a retrospective study of a single private institution. After approval by a Research Ethics Committee (registry: 50722521.6.0000.0087, registry number 4.929.536), the medical records of 137 patients with surgically treated JNAs were reviewed and analyzed between February 1978 and December 2020. A total of 41 patients were excluded due to insufficient data, and 96 cases remained. The following information was collected: age, gender, tumor staging, surgical approach, affected side, and outcome.

All patients were submitted to preoperative and postoperative CT scans. Characteristic radiological features confirmed the diagnosis of all case (enlargement of the pterygomaxillary fissure (PMF) associated with anterior arching of the posterior wall of the maxillary sinus). These were staged according to the classification by Andrews et al., 22 which is as follows: type I – tumor limited to the nasopharynx and nasal cavity, bone destruction negligible or limited to the sphenopalatine foramen; type II – tumor invading the pterygopalatine fossa or the maxillary, ethmoid, or sphenoid sinus with bone destruction; type IIIa – tumor invading the infratemporal fossa or orbital region without intracranial involvement; type IIIb – tumor invading the infratemporal fossa or orbit with intracranial extradural (parasellar) involvement; type Iva – intracranial intradural tumor without infiltration of the cavernous sinus, pituitary fossa or optic chiasm; and type IVb – intracranial intradural tumor with infiltration of the cavernous sinus, pituitary fossa, or optic chiasm.

The surgical treatment consisted of internal and external approaches, using the microscopic technique (transmaxillary and transnasal approach) from 1978 to 1997; and the endoscopic technique (transnasal occasionally associated with transoral and/or transmaxillary approach) from 1997 to 2020. During both periods, a combined approach that consisted of a transoral sublabial access (Caldwell-Luc technique) associated with the microscopic or endoscopic techniques was used whenever necessary. A 30° rigid endoscope was mainly used for the endoscopic technique, with a 45° rigid endoscope used as needed. The centripetal technique 23 was applied in all cases.

The average patient follow-up was of five years, and it consisted of clinical and physical evaluations, endoscopic examinations, and complementary imaging analyses in the immediate postoperative period, 6 months, and 18 months after the surgery. In the past 15 years, follow-up consisted of annual MRI scans 24 months after surgery for a minimum of 5 years.

The data was submitted to a descriptive analysis using a Microsoft Excel 2016 (Microsoft Corporation, Redmond, WA, United States) spreadsheet that contained data on age, affected side, outcome, tumor classification, surgical technique, and transfusion.

## Results

A total of 137 patients with JNA were diagnosed and treated at IFO between 1978 and 2020, and 41 patients with missing data were excluded. All patients were male, with an average age of 17 (range: 10 to 31; median: 16) years.

The tumors were staged as type I in 29 cases (30,2%); type II in 50 cases (52,1%); type IIIa in 12 cases (12,5%); type IIIb in 4 cases (4.2%); and type IVa in 1 case (1.0%). The predominant tumor stage was type II, with 50 cases (52.1%), and 49 patients (51.0%) presented with right nasal tumors ( Table 1 ).

**Table 1:** Andrews et al. 22 classification

Classification	Number of patients
I	29
II	50
IIIa	12
IIIb	4
IVa	1
IVb	0
Total	96

In total, 32 (33.9%) patients underwent the microscopic technique (from 1978 to 1997; 31 transmaxillary approaches and 1 transnasal approach), and 64 (66.7%) were submitted to the endonasal technique (from 1997 to 2020); the combined approach was used in 6 patients (6.3%).

The rate of intraoperative blood transfusion was of 17.7% (17 patients) ( Table 2 ). These patients presented with advanced tumor stages. A total of 13 (70.6%) patients had type-IIIa tumors; 12 (23.5%), type-IIIb tumors; and 1 (5.8%), a type-IVa tumor. The rate of transfusion was of 15.7% for the microscopic approach, and of 18.7% for the endoscopic technique.

**Table 2:** Procedures performed in the present series

Procedures	Patients: n (%)	
Transfusion	17 (17.7)	
Endonasal	64 (66.7)	
Microscopic	32 (33.3)	
Combined	6 (6.3)	

Observations in the follow-up revealed that 2 (2.0%) patients had a residual tumor on the immediate postoperative CT scan. The postoperative complications consisted of blood loss without the need of transfusion and transitional paresthesia. During the follow-up, there were no deaths or other major complications.

## Discussion

Juvenile nasopharyngeal angiofibroma is a benign tumor that originates in vessels located in the PMF. It is locally invasive and can spread along and through natural fissures and foramina of the skull base. 3 5 7 8 11 14 15 24 25 Our observations indicate involvement of the prepterygoid area by the tumor in all cases. This region comprises the anterior portion of the medial pterygoid lamina, in which the pterygoid and pterygopalatine canals are formed.

**Fig. 1:**
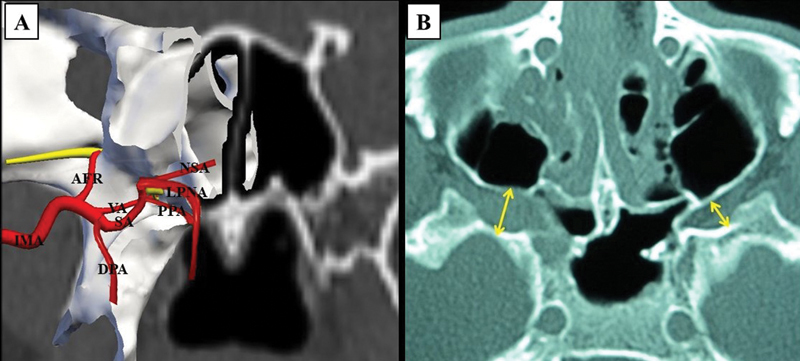
Basic anatomy. ( **A** ) Coronal schematic image of vascularization of the PMF generated with the Nasal Vista (Instituto Tecnológico Nasal, Madrid, Spain) software. ( **B** ) Axial sinus CT scan demonstrating the PMF (arrows) with emphasis on the right-side showing evidence of the enlargement of the PMF (larger arrow). Abbreviations: AFR, artery of the foramen rotundum; DPA, descending palatine artery; LPNA, lateral posterior nasal artery; MA, maxillary artery; PMF, pterygomaxillary fissure; SA, sphenopalatine artery; NSA, nasoseptal artery; PPA, pterygopalatine artery; VA, vidian artery.

The main source of arterial irrigation is the ipsilateral internal maxillary artery (IMA) 3 7 10 26 and its terminal branches, such as the sphenopalatine, nasoseptal, pterygopalatine, and lateral posterior nasal arteries ( Fig. 1A ). Blood supply to JNAs can be highly variable, with collaboration of multiple branches of the external carotid artery (ECA), contralateral ECA branches, and small branches arising from the internal carotid artery (ICA). 27 28 After thorough evaluation of clinical, imaging, and intraoperative data, we believe that the ICA system rarely contributes to tumor nutrition. Dilation of vessels that belong to the ICA system can be explained due to high blood flow inside the tumor, which dilates the regional vessels. It is reasonable to suggest that small branches that arise from ICA collaborate to the complexity of the tumor, but this does not determine the origin of the tumor.

The present study is composed of an exclusively male population with ages ranging from 10 to 31 years, which is in line with other studies. 1 2 3 4 5 6 7 8 9 10 17 19 24 26 Although it is well-known that JNA affects mostly male adolescents, 3 7 10 18 24 29 30 diagnostic suspicion should also be raised in older patients that present with symptoms suggestive of the disease. In two other studies, the ages ranged from 10 to 61 years 31 and from 9 to 74 years, 17 which shows that, although rare, this type of tumor can also affect patients in older age groups. 32

The diagnosis of JNA depends on clinical and imaging evaluations with contrast-enhanced sequences such as CT and MRI scans; CT scans normally reveal an important lesion marker: the anterior displacement of the posterior wall of the maxillary sinus due to enlargement of the pterygopalatine fossa. 24 33 Computed tomography is best used to determine bony changes, and MRI, to determine soft tissue involvement and vascularity, 34 35 specially regarding intracranial extension. Imaging, more specifically contrasted CT, plays an essential role in the diagnosis, assessment of tumor extension, and surgical planning for JNAs; 9 occasionally, angio-CT could be performed.

Several staging systems for JNAs have been proposed. 5 7 8 36 37 38 39 40 41 42 Although there is not a single universally-adopted classification system, 43 the three most prevalent are those by Andrews et al. 22 (modified Fisch), Chandler et al., 40 and Radkowski et al. 39 They all have prognostic value and connotation of surgical approach that have been validated in the era of the open surgical technique. The staging system used in the present article was the one validated by Andrews et al. 22 We recognize that this classification system has limitations, since it does not consider more recent advances in radiologic imaging and surgical techniques. 38

**Fig. 2:**
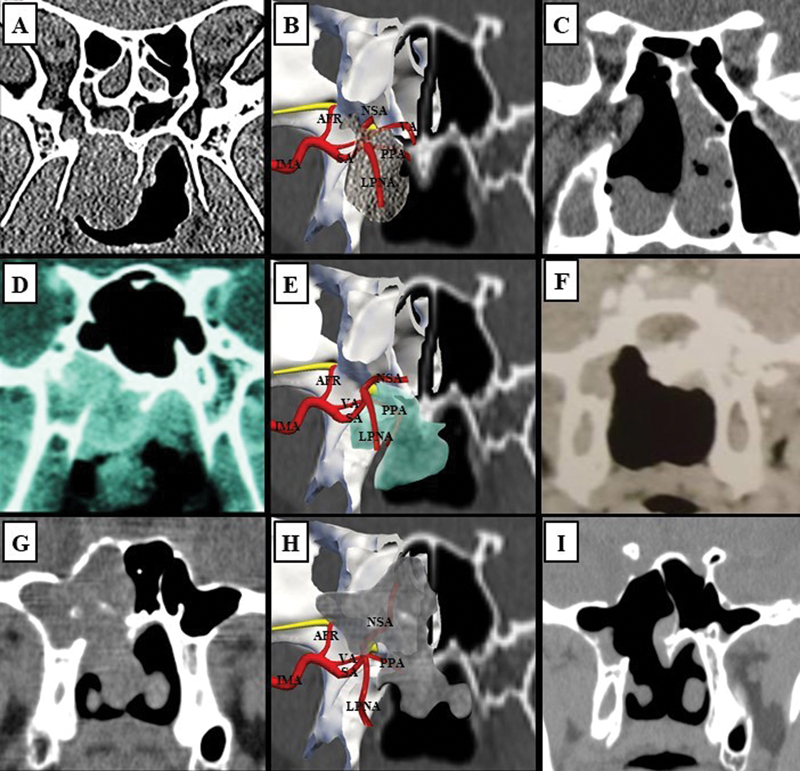
Coronal sinus CT scans of early-stage JNAs and schematic images of anatomical structures of the PMF generated through the Nasal Vista (Instituto Tecnológico Nasal) software. ( **A** ) Type-I JNA on the right nasal side. ( **B** ) Transposition of the tumor to the schematic image demonstrating that the tumor grew down towards the nasopharynx. ( **C** ), Postoperative CT scan. ( **D** ) Type-I JNA on the right nasal side. ( **E** ) Transposition of the tumor to the schematic image demonstrating that the tumor grew down towards the nasopharynx. ( **F** ) Postoperative CT scan. ( **G** ) Type-II JNA on the right nasal side. ( **H** ) Transposition of the tumor to the schematic image demonstrating that the tumor grew up towards the sphenoid sinus, and down towards the nasopharynx. ( **I** ) Postoperative CT scan. Abbreviations: AFR, artery of the foramen rotundum; CT, computed tomography; IMA, internal maxillary artery; JNA, juvenile nasopharyngeal angiofibroma; LPNA, lateral posterior nasal artery; NSA, nasoseptal artery; PPA, pterygopalatine artery; PMF, pterygomaxillary fissure; SA, sphenopalatine artery; VA, vidian artery.

In the present series, there was a higher prevalence of early-stage JNAs, which favored observation and speculation about the origin and expansion of the tumor. ( Fig. 2A-I ). Although some authors attribute the anatomic site of origin to the nasopharynx, 11 44 we agree with Wylie et al. 25 that it arises from the vessels in the sphenopalatine foramen. ( Fig. 1A ). Enlargement of the region formed by the perpendicular plate, sphenoidal apophysis, and orbital apophysis of the palatine bone, as well as the pterygoid bone, was detected in all our cases ( Fig. 1B ).

Imaging analyses and intraoperative observations have revealed that tumor growth and the direction of the propagation are intimately related to the path of least resistance. Additionally, our data corroborates the findings of Antonelli et al., 29 which there are three main routes for intracranial invasion: 1) from the infratemporal fossa, via erosion of the floor of the middle intracranial fossa; 2) via the PMF, next to the superior and inferior orbital fissures; and 3) via the upper wall of the sphenoid sinus, reaching the cavernous sinus and/or pituitary fossa.

**Fig. 3:**
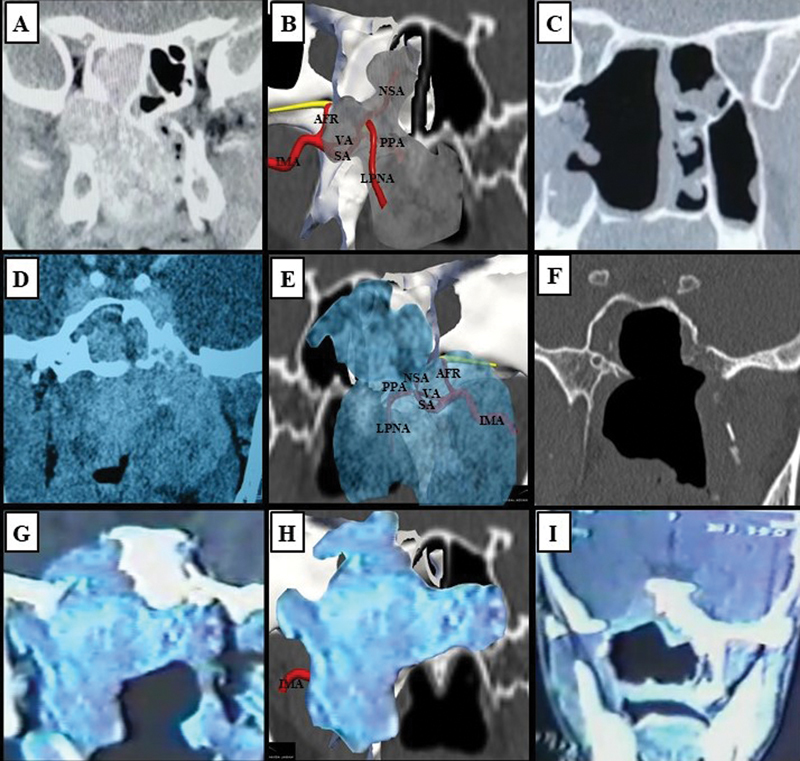
Coronal sinus CT scans of late stage JNAs and schematic images generated through the Nasal Vista (Instituto Tecnológico Nasal) software. ( **A** ) Type-IIIa JNA on the right nasal side. ( **B** ) Transposition of the tumor to the schematic image. ( **C** ) Postoperative CT scan. ( **D** ) Type-IVa JNA on the right nasal side. ( **E** ) Transposition of the tumor to the schematic image. ( **F** ) Postoperative CT scan. ( **G** ) Type-IVb JNA on the right nasal side. ( **H** ) Transposition of the tumor to the schematic image. ( **I** ) Postoperative CT scan. Abbreviations: AFR, artery of the foramen rotundum; CT, computed tomography; IMA, internal maxillary artery; JNA, juvenile nasopharyngeal angiofibroma; LPNA, lateral posterior nasal artery; NSA, nasoseptal artery; PPA, pterygopalatine artery; SA, sphenopalatine artery; VA, vidian artery.

When it comes to larger tumors, they are supplied by multiple vessels as rich collateral irrigation ( Fig. 3A-I ). Additionally, larger tumors can be locally aggressive due to the compressive effect on neighboring structures, and they usually do not invade the dura-mater. In fact, in the 96 cases herein analyzed, only 1 patient (1.0%) presented with intradural invasion, corroborating the current literature. 34

Due to its vascular nature, life-threatening epistaxis and massive intraoperative hemorrhages are a concern. Artery embolization can be performed to minimize bleeding during surgery and facilitate tumor resection. 1 6 13 16 20 21 Recent technological advances have made this procedure safer and more effective, justifying the continuation of this practice. 1 18 28 Nevertheless, data regarding transoperative blood loss after preoperative embolization in JNA resection surgery is controversial. Although various studies show its effectiveness in reducing blood loss and other advantages, 1 6 12 21 28 34 36 other studies, including a systematic review, fail to demonstrate that intraoperative bleeding in embolized patients is statistically lower when compared with nonembolized patients. 45 46 It is worth mentioning that the data in the current literature is not ideal to draw conclusions, since no randomized controlled trials have been conducted. 44 Complications of arterial embolization can occur in 17% to 25% of the patients submitted to the procedure. 46 47 The most compelling complication is migration of an embolus into the intracranial circulation: although rare 21 its significance and morbidity are relatively high.

Despite the advantages of embolization, there are reports that it makes it more difficult to detect nourishing vessels for correct ligation. It hinders tumor dissection as it compromises the accurate identification of tumor margins and achievement of the plane of dissection, since it increases the fibrous component of the tumor. 9 Several studies 48 report that embolization increases the risk of recurrence, especially in cases of deep invasion of the sphenoid bone, because it makes total resection harder to accomplish. Although we recognize the possible advantages of this conduct, we present a series of cases in which JNAs were adequately resected without preoperative embolization.

The gold-standard treatment for JNA is surgical excision of the tumor, 2 7 11 15 16 17 48 which includes external approaches, such as the transpalatine approach, medial maxillectomy (degloving or lateral rhinotomy), maxillary swing, Le Fort osteotomy, infratemporal fossa approach, and endoscopic techniques. 1 2 5 7 11 14 17 44 48 49 The choice of the approach should be based on the stage, site, extension of the lesion, and surgery experience. 1 7

The main advantage of the endoscopic approach is the possibility of obtaining a magnified view of the lesion and related anatomical structures from multiple angles, enabling a better identification of the interface between the lesion and soft tissues or adjacent bone structures, thus also enabling a more accurate and complete dissection and a better control of bleeding. 4 44 48 Other advantages include better exposure, providing a good visualization of lateral or very deep areas, such as the clivus, the foramen lacerum, the roots of the pterygoid, or the infratemporal fossa. Additionally, the avoidance of external incisions, soft tissue detachment, and anterior skeletal osteotomies is probably associated with reduced pre- and postoperative morbidity. 1 44 The primary disadvantage of the endoscopic approach is restricted access and difficulty in shifting to an alternative approach if there is excessive bleeding. Moreover, the availability of expensive instrumentation and extensive endoscopic skull base team training, including the primary and assistant surgeons, can be limiting factors.

Open procedures share the need to perform oral or facial incisions and the need to remove or divide bone to gain access to the tumor, which can result an anesthetic scar and/or facial growth disturbances. 1 17 44 48 49

In our practice, the microscopic technique was used from 1978 to 1997. These procedures were performed before the implementation of endoscopic surgery. After 1997, with the technological development and the advances in high-definition imaging, we shifted to the endoscopic technique, which presents various advantages 44 48 whether alone for intranasal and intraoral accesses, or combined with the transoral sublabial approach (Caldwell-Luc technique). It is worth mentioning that the endoscopic technique with the intraoral approach is especially useful to dissect the posterior boundary of the nasopharynx, which we believe is a critical step in JNA resection. Additionally, when there is the need for a more lateral tumoral access, we prefer the transoral sublabial access (Caldwell-Luc technique) with endoscopic-assisted tumor dissection rather than the Denker approach. A modified Denker approach has been proposed, since it enables the surgeon to achieve extensive exposure of the sinuses and control of the sphenopalatine and IMAs without the risk of palatal dysfunction, oronasal fistula, or facial scarring. 50

We prioritize the use of rigid angled optics, preferably 30°, since viewing angulation enables a more extensive surgical field 4 44 and, therefore, optimizes endonasal maneuverability of the surgical instruments. 4 23 A change in direction of light, provided by the angled endoscope, determines an oculomotor dissociation that enables the surgeon to position the rigid endoscope far from the operating field, which provides a larger area to handle the surgical instruments. This is highly advantageous when dealing with circumstances in which there is bleeding.

The main complication documented in the present series was intraoperative bleeding. The technique used for tumor extraction did not play a significant role in this outcome. According to the data of the present study, tumor stage is more likely to determine intraoperative bleeding, since all patients submitted to blood transfusion presented tumors in advanced stages.

Surgical resection of JNAs is challenging, especially in cases of tumors in advanced stages; therefore, we emphasize the importance of a broader acknowledgment of various surgical approaches and techniques when treating this disease. Our focus is on the surgical technique itself with precise dissection of the tumor boundaries, and we preferably do not use the piecemeal procedure, regardless of the approach adopted. This is especially important when dealing with highly-vascularized tumors, such as JNAs. We agree with Harrison 30 that care should be taken to avoid direct trauma to the tumor. In our observations, manipulation of the tumoral mass predisposes it to intraoperative bleeding. The centripetal technique 23 provides the means for an adequate dissection, far from the tumoral boundaries, and ligation or cauterization of the nourishing vessels, which are key to reducing this outcome, since direct handling of the tumor is avoided. We share a similar methodology to the surgical approach described by Janakiram et al., 9 in which a meticulous dissection of the tumor using the centripetal technique 23 is executed, and distal vascular control is obtained by ligating the branches of the IMA that feed the tumor, reducing intraoperative blood loss. We systematically proceed this way with JNAs.

Total tumor removal was accomplished in 94 cases (98.0%). Immediate postoperative images showed residual tumors in 2 patients (2.0%). Since they remained asymptomatic, a watch-and-wait conduct was adopted with endoscopic and imaging evaluations every six months or if symptoms developed.

We believe that the best technique should guarantee maximal tumor extraction with the least amount of intraoperative bleeding, regardless of the approach adopted, whether endoscopic, external, or combined. Therefore, the choice of surgery should be made without discrimination regarding one approach or another. Factors such as tumor characteristics, patient comorbidities, and expertise of the surgical teas are taken into consideration to make the best decision. It is worth mentioning that team experience and competence with any technique increase over time and affects patient outcomes. 17

The rate of intraoperative transfusion was of 17.7% (17 patients), and it was mainly performed in patients with more advanced tumors. Our observations suggest that tumor vascularization increases as the tumor stage increases, and even after ligation of possible nourishing vessels, the chance of intense intraoperative bleeding is high due to rich collateral angiogenesis.

An obvious weakness of the present study lies in its retrospective nature, although we present a large series. Our data demonstrate total resection in 94 tumors and the need for transfusion in 17 patients. We also acknowledge the lack of minor complication rates. Nevertheless, blood loss requiring transfusion is well documented as a major complication.

We understand that vascular control obtained by ligation or cauterization of vascular branches feeding the tumor is adequate to reduce massive blood loss and surpasses the risk of severe neurologic damage. It is already known that preoperative embolization can be avoided in small tumors, contrary to the common belief that it is indispensable. 9 The present study goes beyond this concept, since we have demonstrated that it is also possible to resect tumors of different stages without previous embolization and with satisfactory patient outcomes.

## Conclusions

The present study demonstrates that resection of JNAs in various stages is viable without previous artery embolization. The centripetal technique enables thorough dissection around the tumor boundaries, which reduces intraoperative bleeding.
